# Preparation and Application of a Novel Liquid Oxygen-Compatible Epoxy Resin of Fluorinated Glycidyl Amine with Low Viscosity

**DOI:** 10.3390/polym16192759

**Published:** 2024-09-29

**Authors:** Jianing Wei, Jia Yan, Shichao Li, Juanzi Li, Zhanjun Wu

**Affiliations:** 1 School of Mechanics and Aerospace Engineering, Dalian University of Technology, Dalian 116024, China; 2 School of Materials Science and Engineering, Dalian University of Technology, Dalian 116024, China; 3 School of Fiber Engineering and Equipment Technology, Jiangnan University, Wuxi 214122, China

**Keywords:** fluororesin, epoxy resin, liquid oxygen-compatible resin, composites tank

## Abstract

A liquid oxygen-compatible epoxy resin of fluorinated glycidyl amine (TFEPA) with a low viscosity of 260 mPa·s in a wide range of temperatures, from room temperature to 150 °C, was synthesized and used to decrease the viscosity of phosphorus-containing bisphenol F epoxy resins. And the forming process and application performances of this resin system and its composite were investigated. The viscosity of the bisphenol F resin was decreased from 4925 to 749 mPa·s at 45 °C by mixing with 10 wt.% TFEPA, which was enough for the filament winding process. Moreover, the processing temperature and time windows were increased by 73% and 186%, respectively. After crosslinking, the liquid oxygen compatibility was preserved, and its tensile strength, elastic modulus, and breaking elongation at −196 °C were 133.31 MPa, 6.59 GPa, and 2.36%, respectively, which were similar to those without TFEPA. And the flexural strength and modulus were 276.14 MPa and 7.29 GPa, respectively, increasing by 21.73% in strain energy at flexural breaking, indicating an enhanced toughness derived from TFEPA. Based on this resin system, the flexural strength and toughness of its composite at −196 °C were 862.73 MPa and 6.88 MJ/m^3^, respectively, increasing by 4.46% and 10.79%, respectively.

## 1. Introduction

In recent years, structural mass has become more and more important in the aerospace field with the development of low-cost and reusable launch vehicles [1,2]. The cryogenic tank, accounting for 40–60% of the total structural dry mass, is the largest component of a launch vehicle [3]. It is becoming increasingly difficult for metal materials to meet the stringent weight reduction requirements of heavy rockets due to their higher density. With carbon fiber reinforced polymer (CFRP) composite materials instead of traditional aluminum alloy, the weight of cryogenic tanks can be reduced by 20–40% [4,5]. However, research on composite cryogenic storage tanks stays at the experimental stage at present, and most of the cryogenic tanks of launch vehicles are still manufactured using metal materials [6,7,8]. The reason for the lack of practical application of composite liquid oxygen cryogenic tanks is mainly due to the compatibility of the resin matrix with liquid oxygen. The term “compatible with liquid oxygen” is used to describe the situation in which a material remains thermal and chemically stable when it is subjected to external loads such as vibration, friction, and impact in liquid oxygen. Most polymers are incompatible with liquid oxygen due to the low temperature and strong oxidizing properties of liquid oxygen. An epoxy matrix is susceptible to combustion and explosion under impact in a liquid oxygen environment, which may have catastrophic consequences. Investigations by the National Aeronautics and Space Administration (NASA) have shown that a large percentage of oxygen-related accidents are caused by incompatibility of materials with liquid oxygen [9]. Therefore, it is necessary to ensure that the epoxy matrix is compatible with liquid oxygen before CFRP composites are used in the manufacture of liquid oxygen cryogenic tanks.

There are many methods to improve the compatibility of epoxy resin with liquid oxygen, and the commonly used method is to add bromide and phosphide to the epoxy system. Wu et al. [10] modified diglycidyl ether of bisphenol A (DGEBA) by adding bromide tetrabromobisphenol A (TBBPA), and this resin system is compatible with liquid oxygen. Nevertheless, in order to reach this high compatibility with liquid oxygen, a large amount of bromide was added, and the viscosity of the resin system was very high. Li et al. [11,12] prepared a series of modified epoxy resins by introducing DOPO or 10-(2,5-dihydroxyphenyl)-9,10-dihydro-9-oxo-10-phosphophenanthrene-10-oxide (ODOPB). It is proved that these modified epoxy systems have good compatibility with liquid oxygen at a high loading levels of phosphorus content. However, their processabilities decrease severely with the increase of phosphorus content. Especially, their high viscosities make them difficult to satisfy some composite material forming processes, such as the filament winding or resin transfer molding process [13,14]. Adding diluents is usually used to solve this problem. Unfortunately, most common diluents, such as dibutyl phthalate (DBP) or butyl glycidyl ether (BGE), are incompatible with liquid oxygen, and as a result make epoxy resin sensitive to liquid oxygen again. At present, few efforts on diluents which are compatible with liquid oxygen and suitable for epoxy resin have been reported.

At present, epoxy resins compatible with liquid oxygen are mainly glycidyl ether-type epoxy resins, which is one of the reasons for their high viscosity. In general, the viscosity of glycidyl amine-type epoxy resins is lower than that of the commonly used glycidyl ether-type epoxy resins, which makes this type of resin have better fluidity and operability in applications [15]. Furthermore, the crosslinked epoxy resin of glycidyl amine always have excellent mechanical properties, heat resistance, and corrosion resistance, and these advantages give them a wide range of potential applications in industrial manufacture. Therefore, it is necessary to synthesize an epoxy resin of fluorinated glycidyl amine with low viscosity and compatibility with liquid oxygen as a diluent to improve the processing properties of the epoxy system used for composite matrixes. Since this fluorinated epoxy resin is already compatible with liquid oxygen, it will not cause the problem of compatibility with liquid oxygen when it is added to a resin system that is already compatible with liquid oxygen. Moreover, this fluorinated epoxy resin has a similar structure to other epoxy resins, so there is no problem of miscibility between them [16]. Up to now, few efforts have been made about the compatibility of thermosetting fluorinated epoxy resin with liquid oxygen [1,17].

In this paper, a thermosetting epoxy resin of fluorinated glycidyl amine, 3-(trifluoromethyl)-*N*,*N*-bis(2,3-epoxypropyl)aniline (TFEPA), was synthesized from epichlorohydrin (ECH) and 3-(trifluoromethyl) aniline (m-TFMA). Then, it was crosslinked by 4,4′-diaminodiphenylmethane (DDM) as a curing agent. The chemical structure and viscosity of the TFEPA monomer were discussed, and the curing behavior, compatibility with liquid oxygen, and thermal properties of the crosslinked TFEPA epoxy resin were tested and analyzed. In our previous study, an aryl phosphinate diglycidyl ether, 10(2′,5′-bis(glycidyloxy)phenyl)-9,10-dihydro-9-oxa-10-phosphaphenanthrene-10-oxide (PDGEP), was verified to have the ability to improve the compatibility of a resin matrix with liquid oxygen [18]. However, due to the high viscosity of PDGEP, the epoxy resin system with the addition of PDGEP makes it difficult to achieve the viscosity requirements of composite molding processes with low viscosities, such as the filament winding process. Therefore, TFEPA was added as a reactive diluent to the PDGEP-modified epoxy system of diglycidyl ether of bisphenol F (DGEBF) to improve its processing properties in this study. The addition of TFEPA was tested for its effects on the processing properties, curing behavior, liquid oxygen compatibility, thermal properties, and mechanical properties of the epoxy system. Finally, the mechanical properties of CFRP composites with or without the addition of TFEPA at −196 °C were tested and discussed.

## 2. Materials and Methods

### 2.1. Materials

ECH, m-TFMA, sodium methoxide solution (30% wt), and DDM were purchased from Shanghai Macklin Biochemical Co., Ltd., Shanghai, China. ODOPB was purchased from Huizhou Sunstar Technology Co. Ltd., Huizhou, China. Deuterated chloroform (CDCl_3_) was purchased from Aladdin-reagent Co., Shanghai, China. Methanol was purchased from Sinopharm Chemical Reagent Co., Ltd., Shanghai, China DGEBF (commercial mark 830) was purchased from Nantong Xingchen Synthetic Material Co., Ltd., Nantong, China. Carbon fiber orthogonal woven fabric (T300) was purchased from Daxing Composites, Yixing, China. All other reagents and solvents were obtained commercially and used as received.

### 2.2. Synthesis of TFEPA Monomer

**Scheme 1:**
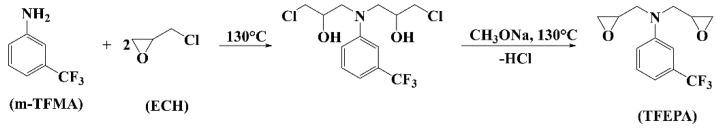
Reaction process of TFEPA monomer.

ECH (185 g, 2 mol) and m-TFMA (32.2 g, 0.2 mol) were mixed in a three-neck flask equipped with a mechanical stirrer, reflux condenser, and temperature controller. The mixture was slowly heated to 130 °C and maintained at that temperature for 8 h. Next, sodium methoxide solution (72 g, 0.4 mol) was added at room temperature. The mixture was heated again to 130 °C and held further for 10 h. After that, the by-product salt was removed by filtration and extraction. And the obtained product was dried and concentrated on a rotatory evaporator under reduced pressure at 130 °C for 4 h to get the final product (46.5 g, yield 85.2%). The preparation procedure is illustrated in Scheme 1.

### 2.3. Synthesis of PDGEP

**Scheme 2:**
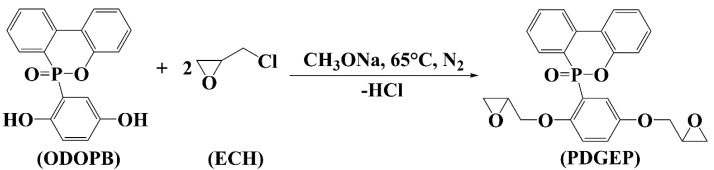
Reaction process of PDGEP.

The PDGEP was prepared successfully according to our previous report [18]. Typically, ECH, ODOPB, and sodium methoxide (molar ratio of 10:1:2) were mixed in a three-necked flask assembled with a mechanical stirrer, reflux condenser, temperature controller, and nitrogen inlet. Then, the mixture was stirred at 65 °C under N_2_ atmosphere for 18 h. After completion of the reaction, the reaction mixture was filtered and then extracted by deionized water for several times. Finally, the separated organic liquid was dried and concentrated on a rotary evaporator under reduced pressure at 130 °C for 6 h to get the homogeneous solid PDGEP. The preparation procedure is illustrated in Scheme 2.

### 2.4. Preparation of Epoxy Matrix

**Scheme 3:**
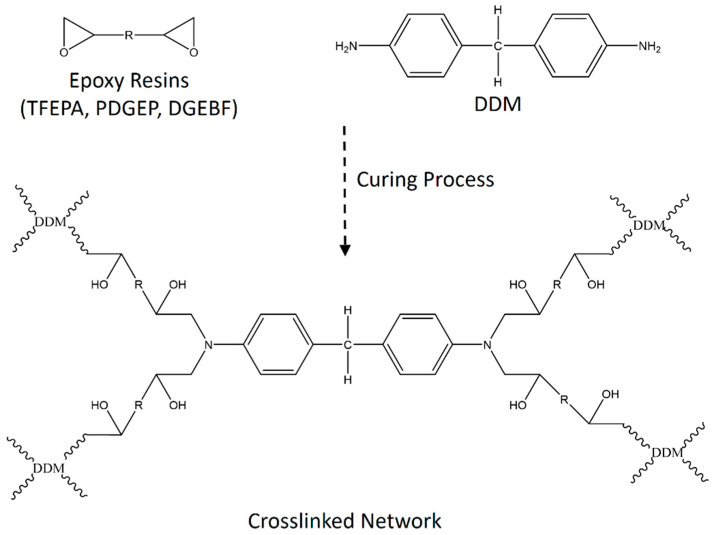
Curing process of epoxy matrixes.

The curing process is the reaction of the amino groups of the curing agent DDM and the oxirane ring of the resins to form a crosslinked network, as shown in Scheme 3.

The crosslinked TFEPA (denoted as FEP) was prepared by the following process: DDM was used as a curing agent, and it was blended with TFEPA monomer at a molar ratio of N–H/epoxy group 1:1. The mixture was heated to 80 °C and stirred at that temperature until the DDM was completely dissolved. Then, the received mixture was degassed in a vacuum oven to remove bubbles at the same temperature for about 5 min and poured into Teflon molds immediately. Finally, the mixture was transferred to an air-circulating oven, followed by a step-curing process at 110 °C for 2 h, 150 °C for 2 h, and 180 °C for 2 h.

TFEPA and PDGEP-modified DGEBF resin systems were prepared by the following process: TFEPA and PDGEP were dissolved in DGEBF at 100 °C with constant stirring until a homogeneous epoxy resin was obtained. Then, an appropriate amount of DDM (molar ratio of epoxy groups to N-H is 1:1) was added at 80 °C, and the mixture was stirred until DDM was completely dissolved. Finally, the mixture was completely degassed under a vacuum oven and poured into preheated molds to cure using the following procedure: first, at 90 °C for 2 h, then at 130 °C for 2 h, and finally at 170 °C for 2 h. Since previous work [18] had proved that 10% of PDGEP was the optimal ratio, the content of PDGEP was selected as 10%. In order to compare the effect of the diluent amount on the viscosity of the resin, the three different mass ratios of TFEPA were designed, in which the amount of TFEPA resins were added exponentially. The epoxy matrixes in the mass ratio of TFEPA:PDGEP:DGEBF are 1:1:8, 2:1:7, and 4:1:5 and were denoted as EP1, EP2, and EP3, respectively. As a control, the epoxy matrix without TFEPA (PDGEP:DGEBF 1:9) was also prepared by the same method with a curing process of 90 °C for 2 h, 130 °C for 2 h, and 170 °C for 2 h (denoted as EP0).

### 2.5. Preparation of Carbon Fiber-Reinforced Epoxy Matrix Composites

The vacuum-assisted infusion process was used to prepare the carbon fiber-reinforced EP1 epoxy composites (denoted as CF/EP1). The system was assembled with the following materials from bottom to top: heating plate; peel ply; 9 plies of dry carbon fiber fabrics; peel ply; infusion mesh; release film and vacuum bag. The heating plate and vacuum bag were glued together with sealed tape to maintain the low vacuum of the system. The appropriate amount of EP1 was prepared and degassed in a vacuum oven at 80 °C as the resin matrix. The resin matrix was infiltrated with dry carbon fiber fabrics by an infusion tube. The system was cured with a vacuum infusion technique by the following process: 90 °C for 2 h, 130 °C for 2 h, and 170 °C for 2 h. The EP0 was also used to prepare the carbon fiber-reinforced epoxy composites (denoted as CF/EP0) with the same curing process for comparison.

### 2.6. Characterization

Fourier transform infrared spectroscopy (FTIR) of the TFEPA monomer and m-TFMA were performed using a ThermoFisher 6700 spectrometer (Thermo Fisher Scientific, Waltham, MA, USA) with a resolution of 4 cm^−1^ in the wavenumber range from 4000 cm^−1^ to 500 cm^−1^.

^1^H, ^13^C, and ^19^F nuclear magnetic resonance (NMR) spectra were recorded on a Brucker AVANCE I500 (Brucker, Basel, Switzerland). The sample was 46 mg/mL, with CDCl_3_ as solvent.

Liquid chromatography-mass spectrometry (LC-MS) analysis was obtained on Q Exactive (Thermo Fisher Scientific, Waltham, MA, USA). The system was operated in positive ionization mode. And the sample was dissolved in methanol and deionized water.

The epoxy value was determined by the hydrochloric acid acetone method, according to the national standard GB/T 1677-2008 [19].

The rheological properties of the samples were determined by DHR-2 rheometer (TA Instruments, New Castle, DE, USA). The heating rate and temperature range were set to 2 °C/min and from 30 °C to 150 °C for the rheological measurements of epoxy samples. An oscillation-temperature ramp measurement was used for the rheological measurements of epoxy resins with curing agent at a heating rate of 2 °C/min from 30 °C to 150 °C. The isothermal oscillation viscosity method was adopted at 50 °C for 3 h. All the above tests were conducted using a frequency of 1 Hz and a constant strain of 0.1%. Epoxy resins and the curing agent were mixed before testing, and all of the testing began after the curing agent was completely dissolved.

The compatibility of the different epoxy systems with liquid oxygen were tested by mechanical impact in ambient liquid oxygen on an ABMA-type impact tester, as described in ASTM G86-17 [20]. This standard described test equipment and techniques to determine the impact sensitivity of materials in ambient pressure liquid oxygen, and the detailed test steps are as follows: Firstly, a sample of epoxy resin is placed in a sample cup filled with liquid oxygen, and then a striker pin is centered in the cup. Secondly, a 10 kg plummet is dropped from the height of 1 m onto the pin, which transmits the 98 J impact energy to the test sample. Impact sensitivity reactions such as burning, explosion, flash, and charring during the test were recorded. The material was liquid oxygen-compatible if it satisfied one of the following conditions: (1) no incompatible reactions for 20 samples tested at the 98 J energy level; (2) a maximum of one incompatible reaction in 60 samples tested at the 98 J energy level.

Differential scanning calorimetry (DSC) was conduct using a Q2000 analyzer instrument (TA Instruments, USA). In order to measure the glass-transition temperature (T_g_), the samples were heated at a rate of 20 °C/min from 40 °C to 180 °C and the temperature held for 5 min, then it was reduced to 100°C and held for 5 min, and finally it was increased to 240 °C. As for non-isothermal curing kinetic tests, epoxy resins and the curing agent were heated at different heating rates of 5 °C/min, 10 °C/min, 15 °C/min, and 20 °C/min from 50 °C to 250 °C.

Thermogravimetric analysis (TGA) and differential thermogravimetric (DTG) were conducted using a Q500 analyzer instrument (TA Instruments, USA) under nitrogen atmosphere. The samples were heated at different heating rates (5 °C/min, 10 °C/min, 15 °C/min, 20 °C/min) from 50 °C to 800 °C.

Dynamic mechanical analyses (DMAs) of epoxy matrixes were conducted using a Q800 instrument (TA Instruments, USA). The samples were heated from 30 °C to 250 °C at a heating rate of 3 °C/min. The measurement was performed by single-cantilever beam mode at a frequency of 1 Hz. And the sample size was 40 × 4 × 3 mm^3^.

Tensile properties of EP0 and EP1 at room temperature (RT) and −196 °C were measured on a microcomputer-controlled electronic universal testing machine with environment chamber (Lishi instruments Co. Ltd., Shanghai, China) according to ASTM D638 [21]. The tensile strain was measured on an extensometer (Epsilon Technology, Jackson, WY, USA). At least 5 experiments were conducted for each group, and the final results were the average of the data.

Flexural properties of the samples were measured on a Servo-Hydraulic Mechanical Tester (Changchun Research Institute for Mechanical Science Co., Ltd., Changchun, China). The cryogenic environment was achieved by spraying liquid nitrogen into an environment chamber (Changchun Fangrui Technology Co., Ltd., Changchun, China). The tests for EP0 and EP1 were performed according to ASTM D790 [22], with a specimen size of 80 × 13 × 4 mm^3^. And the tests for CF/EP0 and CF/EP1 were performed according to ASTM D7264 [23], with a specimen size of 80 × 13 × 2 mm^3^. A three-point loading configuration was used for the experiment, and the support span was 64 mm. At least 5 experiments were conducted for each group, and the final results were the average of the data.

## 3. Results and Discussion

### 3.1. Characterization of TFEPA Monomer

**Figure 1:**
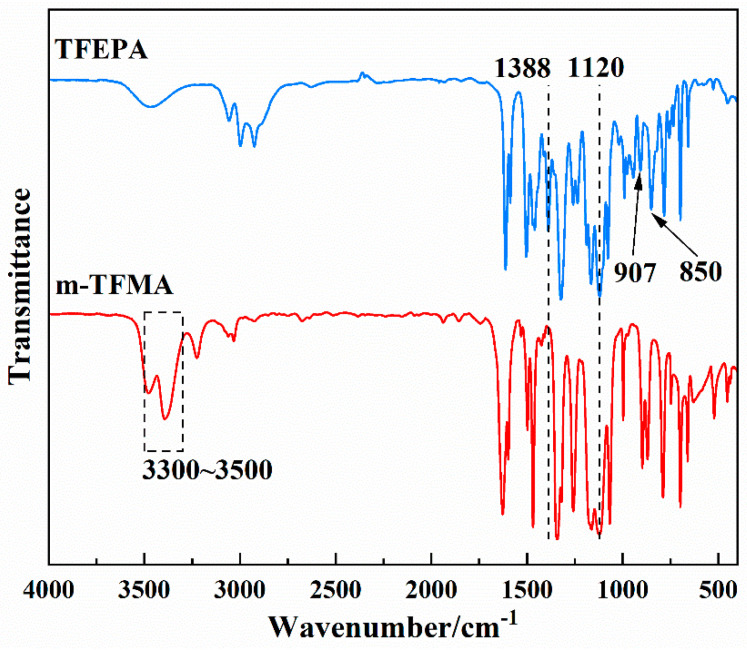
FTIR spectra of the m-TFMA and TFEPA monomer.

The FTIR spectra of the m-TFMA and TFEPA monomer are shown in Figure 1. A strong absorption peak at 1120 cm^−1^ could be attributed to the C–F bond in both spectra. In comparison to the spectrum of m-TFMA, the two medium absorption peaks of primary amine in the range of 3300 cm^−1^ to 3500 cm^−1^ disappeared, while the absorption peak of C–N bond and oxirane ring vibration appeared at 1388 cm^−1^, 907 cm^−1^, and 850 cm^−1^ in the spectrum of the TFEPA monomer, respectively, which proved that ECH and m-TFMA were sufficiently reacted [24,25,26].

**Figure 2:**
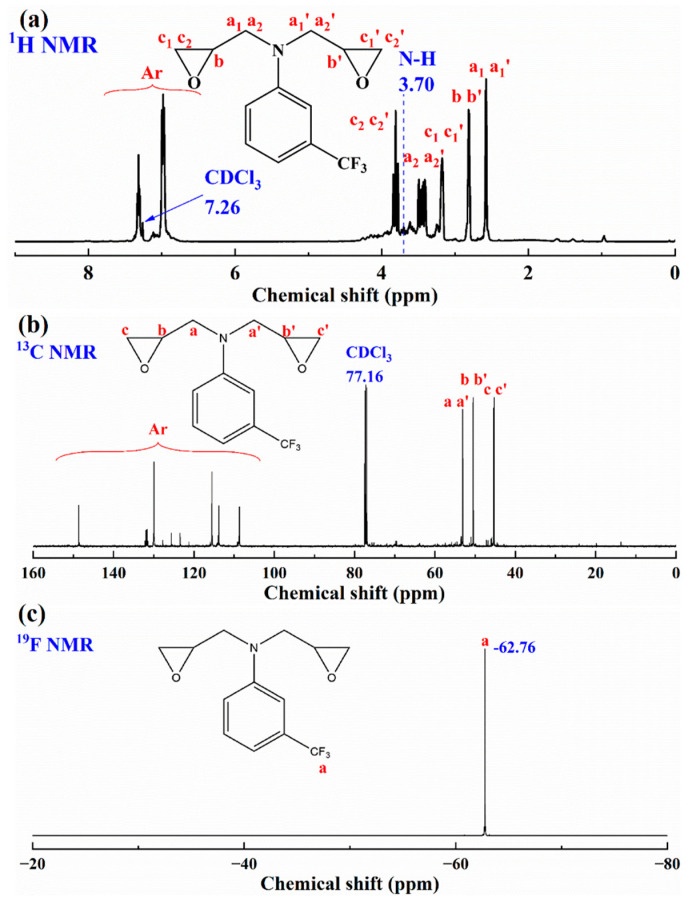
^1^H NMR spectrum (**a**), ^13^C NMR spectrum (**b**), and ^19^F NMR spectrum (**c**) of the TFEPA monomer.

Figure 2a shows the ^1^H NMR spectrum of the TFEPA monomer. The peaks in the range from 6.5 to 7.5 ppm were assigned to the H atoms on the aromatic ring. The peaks in the range from 2.75 to 3.25 ppm and from 3.75 to 4 ppm were assigned to the H atoms of oxirane rings. The chemical shift of H atoms belonging to the eNH_2_ group on the m-TFMA is 3.70 ppm [27]. The absence of a significant peak at 3.70 ppm in the spectrum of the TFEPA monomer indicated the transformation of primary amine to tertiary amine. Figure 2b displays the ^13^C NMR spectrum of the TFEPA monomer. The peaks in the range from 105 to 150 ppm were assigned to the C atoms on the benzene ring and the eCF3 group. The peaks in the range from 42.5 to 52.5 ppm were assigned to the C atoms of oxirane rings. Figure 2c shows the ^19^F NMR of the TFEPA monomer. It is obvious that there was only one signal at −62.76 ppm assigning to the F atoms on the eCF_3_ group. The chemical shift of the F atoms on the m-TFMA was −63.38 ppm [28]. The different chemical shifts of F atoms between m-TFMA and the TFEPA monomer proved that the chemical structure of the TFEPA monomer was different from m-TFMA. These results indicated that the eCF_3_ group was introduced into the epoxy system successfully.

**Figure 3:**
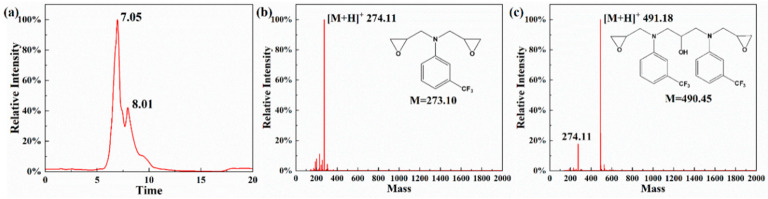
(**a**) The TIC of TFEPA monomer, (**b**) MS spectrum of the peak at 7.05 min, (**c**) MS spectrum of the peak at 8.01 min.

Figure 3a displays the total ions chromatogram (TIC) of the TFEPA monomer by LC-MS. There was a major peak at 7.05 min and a minor peak at 8.01 min. Figure 3b,c displays the MS spectra of the major peak at 7.05 min and the minor peak at 8.01 min, respectively. The chemical formula of the TFEPA monomer was C_13_H_14_F_3_NO_2_, and its exact mass was 273.10. Therefore, it was obvious that the peak at 274.11 *m*/*z* in Figure 3b assigned to the TFEPA monomer acquired a proton under the positive ionization condition [29]. Due to the large site resistance of the benzene ring on the side chain of TFMA, the first step of the reaction was probably insufficient. In this case, the TFEPA monomers might be further polymerized with each other to create the TFEPA polymer [30]. Accordingly, the peak at 491.18 *m*/*z* in Figure 3c was assigned to the TFEPA oligomer with *n* = 1.

The maximum theoretical epoxide value of the TFEPA monomer was 0.73 mol/100 g from the molecular structure. The epoxide value of the obtained TFEPA monomer from the reaction was 0.703 mol/100 g, which was very close to the theoretical epoxy value, suggesting a successful synthesis of epoxy.

### 3.2. Rheology Performance Analyses

The TFEPA monomer was a liquid with good fluidity at RT. As shown in Figure S1, the viscosity of the TFEPA monomer was stabilized at about 260 mPa·s with a range of temperature from RT to 150 °C, indicating that it had a wide processing temperature window. In addition, the viscosity of TFEPA (270 mPa·s at 25 °C) is significantly lower than that of commercial bisphenol F resin (ranging from 2000 to 5000 mPa·s at 25 °C) [31] and bisphenol A resin diluted with styrene (ranging from 900 to 1500 mPa·s at 25 °C) [32], which is close to the viscosity of a reactive diluent (cardanol epoxy acrylates, 170 mPa·s at 25 °C) [33]. Moreover, such low viscosity characteristics can provide good wettability and adhesion to the substrate, which provides good operability in the preparation of composite materials [34].

**Figure 4:**
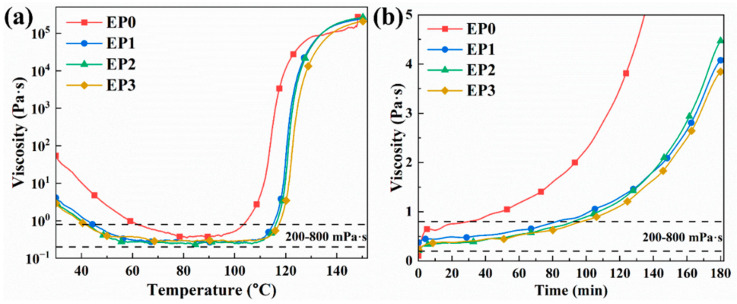
The viscosity vs. temperature curves (**a**) and viscosity vs. time curves at 50 °C (**b**) of different epoxy matrixes.

In order to evaluate the effect of the TFEPA monomer on the processing ability of an epoxy matrix, the rheology performances of EP0, EP1, EP2, and EP3 were investigated. Figure 4a shows the viscosity vs. temperature curves of different epoxy matrixes. It can be seen that the viscosity of all the epoxy matrixes firstly decreased and then increased sharply with the increase of temperature. Different from EP0, the viscosity curves of EP1, EP2, and EP3 (all with TFEPA) developed a plateau over a long temperature range, and their viscosities remained around 300 mPa·s. Figure 4b shows the viscosity vs. time curves at 50 °C of different epoxy matrixes. It is clear that the viscosity of all the epoxy matrixes increased with the increase of time at the same temperature. The filament winding process commonly used in the manufacture of tanks has stringent requirements for the viscosity of the resin matrix; it needs the viscosity to be in the range of 200 mPa·s to 800 mPa·s from 40 °C to 50 °C [13]. At 45 °C, the viscosity of all TFEPA-enhanced resin systems satisfied the filament winding process, with viscosities of 558–749 mPa·s, which were much lower than the 4925 mPa·s viscosity of EP0. Table 1 lists the processing temperatures and processing times that satisfied the filament winding process. As shown in Table 1, the processing temperature window of EP1 was from 44 °C to 115 °C, and the processing time at 50 °C of EP1 was 83 min. Compared to EP0, the processing temperature window and processing time at 50 °C of EP1 increased by 73% and 186%, respectively. Obviously, the addition of TFEPA enhanced the processing temperatures and processing times of the epoxy matrix significantly. Nevertheless, the addition of more TFEPA had little effect on the processing temperatures and processing times of the epoxy matrix. The processing temperature window of EP3 was from 41 °C to 117 °C, which increased by 7% compared to EP1. And the processing time at 50 °C of EP3 was 96 min, which increased by 16% compared to EP1. The effect of lowering viscosity achieved by the addition of more TFEPA was significantly reduced. Therefore, both epoxy matrixes EP2 and EP3 were not considered in subsequent analyses. In conclusion, the epoxy resin TFEPA could be used as a reactive diluent to improve processing properties.

### 3.3. Curing Behavior

**Figure 5:**
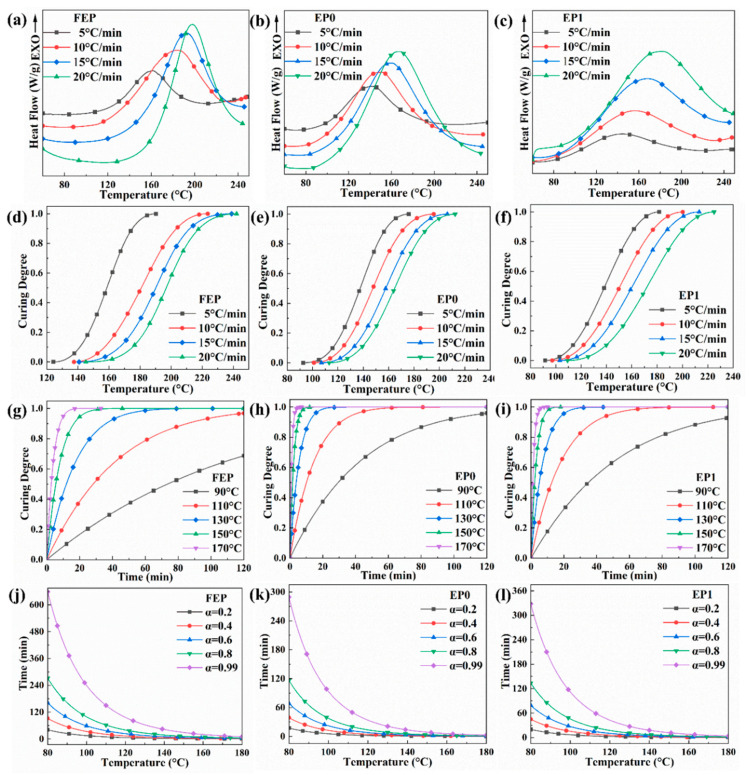
DSC curves at different heating rates (**a**–**c**). Curves of the curing degree and temperature (**d**–**f**). Curves of the curing degree and time at different curing temperatures (**g**–**i**). Curves of the curing temperature and curing time at different curing degrees (**j**–**l**).

The processing properties of the epoxy systems were further investigated by the analysis of curing kinetics, which provided some reference for the curing process of the resin. DSC tests were performed on uncured FEP, EP0, and EP1, respectively, at different heating rates, and the results are shown in Figure 5a–c. The curing process at different heating rates displayed a single excipient peak, which indicated that the curing reaction is a one-step process [35]. Table 2 lists the onset curing temperature (*T_o_*), the peak exothermic temperature (*T_p_*), and the end curing temperature (*T_e_*) for the two epoxy matrixes. The characteristic temperature increased as the rate of heating increased, and the exothermic peak of curing moved towards higher temperatures [36]. The *T_o_*, *T_p_*, and *T_e_* for FEP with a heating rate of 0 °C/min were obtained by extrapolation (Figure S2) as 111.88 °C, 149.24 °C, and 179.04 °C. Therefore, the curing process of FEP was determined as a step process of 110 °C for 2 h, 150 °C for 2 h, and 180 °C for 2 h. Similarly, the curing process of EP0 and EP1 can be determined from Figures S3 and S4 as a step process of 90 °C for 2 h, 130 °C for 2 h, and 170 °C for 2 h.

In addition, the relationship between the curing degree α and the exothermic enthalpy of curing reaction can be obtained from the DSC curve by the following equation [37]:

(1) $$\alpha =\frac{∆H_{t}}{∆H_{total}}$$

where α is the curing degree, $∆H_{t}$ is the exothermic enthalpy of curing reaction at time t, and $∆H_{total}$ is the total exothermic enthalpy of curing reaction. The relationship between the curing degree and temperature of the three epoxy matrixes are shown in Figure 5d–f. With the increase of the heating rate, the system required a higher temperature to reach the same curing degree. This is mainly due to the reaction rate, and that the viscosity of the system increases faster with the increase of the heating rate, with the result that some of the epoxy monomers do not have time to react or diffuse into the gel system.

The non-isothermal curing kinetics were investigated via the Kissinger method [38], Crane method [39], and Borchardt–Daniels method [40]. The equation of the Kissinger method is

(2) $$\ln\frac{\beta }{T_{p}^{2}}=\ln\frac{AR}{E_{\alpha }}-\frac{E_{\alpha }}{RT_{p}}$$

where β is the heating rate, $T_{p}$ is the peak temperature, $E_{\alpha }$ is the apparent activation energy, A is the pre-exponential factor, and R is the universal gas constant (8.314 J·mol^−1^·k^−1^). The reaction order n can be obtained according to the Crane equation,

(3) $$\frac{d\left(\ln\beta \right)}{{d(T}_{p}^{-1})}=-\left(\frac{E_{\alpha }}{nR}+2T_{p}\right)$$

In general, *E_α_*/*n*R ≫ 2*T_p_*, so Equation (3) can be changed to Equation (4) [37],

(4) $$\frac{d\left(\ln\beta \right)}{{d(T}_{p}^{-1})}=-\frac{E_{\alpha }}{nR}$$

Based on Equations (2) and (4), *E_α_*, A and, n of FEP, EP0, and EP1 can be calculated from the linear fit curves of $\ln\left(\beta /T_{p}^{2}\right)$ and ln⁡β vs. 1/$T_{p}$ shown in Figures S5–S10. For FEP, EP0, and EP1, the value of *E_α_* is 55.381 kJ/mol, 61.948 kJ/mol, and 58.822 kJ/mol; the value of A is 8.424 × 10^5^ min^−1^, 1.874 × 10^7^ min^−1^, and 5.578 × 10^6^ min^−1^; and the value of n is 0.88, 0.90, 0.89, respectively.

The Borchardt–Daniels method is based on a single heating rate in order to analyze the curing reaction with n-level kinetics, expressed by Equation (5) and its variant form Equation (6),

(5) $$\alpha =1-{\left[1-\left(1-n\right)Atexp\left(-\frac{E_{\alpha }}{RT}\right)\right]}^{\frac{1}{1-n}}$$

(6) $$t=\frac{{1-(1-\alpha )}^{1-n}}{\left(1-n)Aexp(-\frac{E_{\alpha }}{RT}\right)}$$

By substituting the values of *E_α_*, *A*, and n into Equation (5), the curves of curing degree and time at different curing temperatures can be obtained, as shown in Figure 5g–i. It can be concluded that the curing rate increases with the increase of temperature. When the temperature is lower than *T_o_*, the system cures slowly, and when it is higher than *T_o_*, the curing rate increases dramatically. Figure 5j–l shows the curves of curing temperature and curing time at different curing degrees obtained from Equation (6). It is obvious that with higher curing temperatures, the time required to achieve the same curing degree is shorter. In addition, the EP1 epoxy system had a lower curing degree at the same temperature for the same time than EP0, and the time required to reach the same curing degree at the same temperature was longer, indicating that EP1 had a longer processing time than EP0. Theoretically, by keeping FEP, EP0, and EP1 at *T_p_* for 54 min, 51 min, and 42 min, respectively, the curing degree could reach 1, and the three resin systems would be fully cured. In order to ensure that the three resin systems were fully cured, the curing time of each stage was chosen to be 2 h.

### 3.4. The Compatibility of Epoxy Matrix with Liquid Oxygen

**Figure 6:**
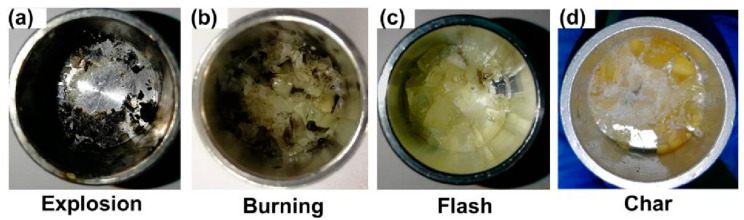
Samples with (**a**) explosion, (**b**) burning, (**c**) flash, and (**d**) char after the liquid oxygen compatibility test.

In this study, the three epoxy matrixes were made into samples with a diameter of 20 mm and a thickness of 2.5 mm. All of the samples were polished with sandpaper and pre-cooled in liquid oxygen for about 10 min before testing. The impact reaction sensitivity (IRS) coefficient was used to evaluate the response intensity between materials and liquid oxygen under mechanical impact. The *IRS* can be calculated by the following formula [41]:

(7) $$IRS=\frac{\sum_{i}^{4}w_{i}n_{i}}{N}$$

where $w_{i}$ is the weight coefficient, in which $w_{1}$ (burning) = 1, $w_{2}$ (explosion) = 0.9, $w_{3}$ (flash) = 0.6, $w_{4}$ (charring) = 0.4; $n_{i}$ is the frequency of sensitivity reactions, and N is the total number of tests. Figure 6 displays this four sensitive reactions of epoxy resins with liquid oxygen.

Table 3 lists the results of the test. There was only one flash in 60 tests for the FEP and no impact sensitivity reaction for either EP0 or EP1. The results indicated that all three epoxy systems passed the test, which proved that they were all compatible with liquid oxygen. Much research has demonstrated that DGEBF is not compatible with liquid oxygen [42,43]. It is evident from the experimental results that the addition of PDGEP reduced the IRS from 17% to 0, transforming the system from incompatible to compatible with liquid oxygen. Moreover, since TFEPA was compatible with liquid oxygen, the addition of TFEPA to the EP0 system did not decrease its compatibility with liquid oxygen, and the EP1 system was still compatible with liquid oxygen.

### 3.5. Thermal Analysis

**Figure 7:**
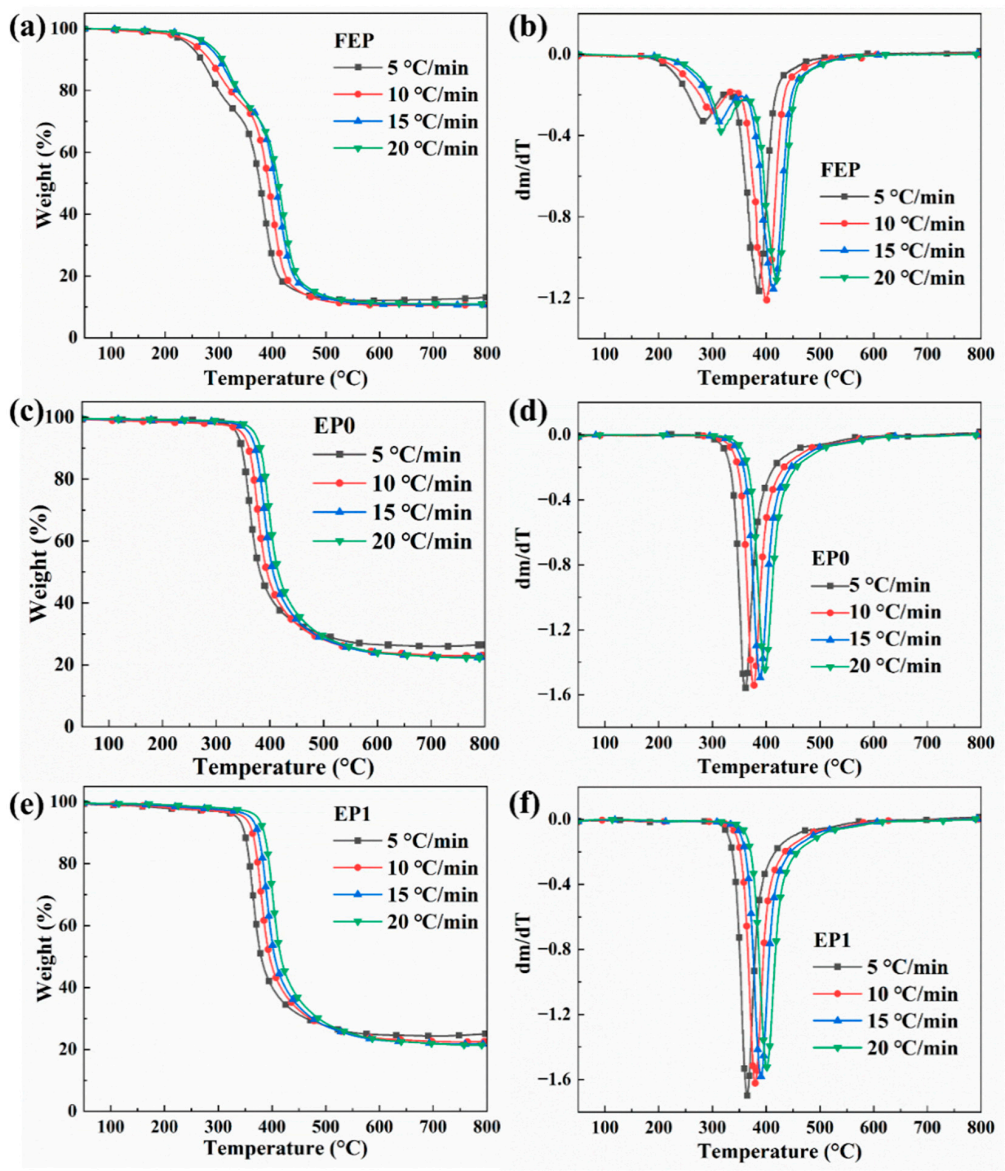
TGA and DTG curves of FEP (**a**,**b**), EP0 (**c**,**d**), and EP1 (**e**,**f**) samples at different heating rates.

The thermal stability of FEP, EP0, and EP1 epoxy matrixes was evaluated by the TGA test. Figure 7 shows the TGA curves and DTG curves of the three samples at different heating rates under nitrogen atmosphere. Table 4 lists the decomposition temperature of 5% weight loss (T_d5%_), the decomposition temperature of 10% weight loss (T_d10%_), the decomposition temperature for the maximum rate of weight loss (T_dmax_), maximum degradation rate, and the carbon residue rate at 800 °C. It was obvious that with the increase of the heating rate, all of the three materials exhibited thermal hysteresis, and the decomposition temperature increased gradually [44]. Moreover, FEP exhibited two decomposition stages, and EP0 and EP1 exhibited one decomposition stage. The first decomposition stage of FEP was the volatilization of the aromatic rings on the branched chain because of the breakage of C–N bonds [30]. The second decomposition stage of FEP and the first decomposition stage of EP0 and EP1 were the decomposition of the epoxy skeleton. The T_dmax_ of FEP (385–421 °C) was higher than that of EP0 (361–398 °C) and EP1 (366–401 °C) due to the C–F bond having higher energy than the C–C bond [45,46]. In addition, the differences in T_d5%_, T_d10%_, T_dmax_, and carbon residue between EP0 and EP1 were small, indicating that the addition of TFEPA had almost no effect on the thermal stability of the epoxy system.

**Figure 8:**
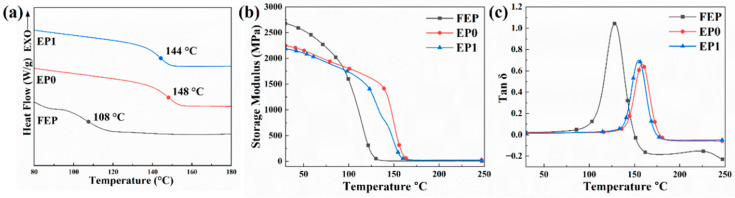
(**a**) DSC curves of FEP, EP0, and EP1 samples, storage modulus (**b**), and tan δ (**c**) vs. temperature curves of FEP, EP0, and EP1 samples.

Figure 8 and Table 5 display the DSC and DMA results of the FEP, EP0, and EP1 samples. As shown in Figure 8b, the storage modulus of the three samples decreased as the temperature increased, which indicated the enhancement of the molecular chain movement. All samples exhibited an obvious transition from the glassy to the rubbery state due to α relaxation of the molecular chains [47]. The storage modulus at 30 °C ($E^{\prime }$) of FEP was 2680.86 MPa, which was higher than that of EP0 (2247.01 MPa) and EP1 (2186.11 MPa). This was mainly attributed to more aromatic rings on the side chain of TFEPA, giving greater rigidity to the structure [36]. As shown in Figure 8c, the tan δ curves of all the samples exhibited only one peak, indicating that all the three resin systems formed a homogeneous crosslinked epoxy network, and the miscibility among TFEPA, PDGEP, and DGEBF was excellent [48]. The trends of T_g(DSC)_ and T_g(DMA)_ remained consistent. As for T_g(DSC)_, the T_g_ of EP0 was 148 °C, which was slightly higher than the 144 °C of EP1, and FEP had a much lower T_g_ of 108 °C. The T_g_ value of epoxy resins is usually related to their chemical structure, intermolecular interactions, and cured crosslinking density [49]. According to the rubbery elasticity theory [50], the crosslinking density (ρ) can be estimated by the following theoretical formula:

(8) $$\rho =\frac{E_{r}^{\prime }}{3RT}$$

where $E_{r}^{\prime }$ is the rubbery modulus, R is the universal gas constant, and $T=T_{g}+30\;(K)$. By calculation, the crosslinking density of FEP, EP0, and EP1 was 671 mol/m^3^, 2014 mol/m^3^, and 2006 mol/m^3^. It could be seen that the addition of TFEPA had essentially no effect on the crosslink density. In addition, the crosslink density of FEP was significantly lower than that of EP0 and EP1, suggesting the reason for its lower *T_g_* [51].

In conclusion, there was little difference between the thermal stability, *T_g_*, and storage modulus of EP0 and EP1. The addition of TFEPA had a little effect on the thermal properties of the epoxy system.

### 3.6. Mechanical Properties of EP0 and EP1

**Figure 9:**
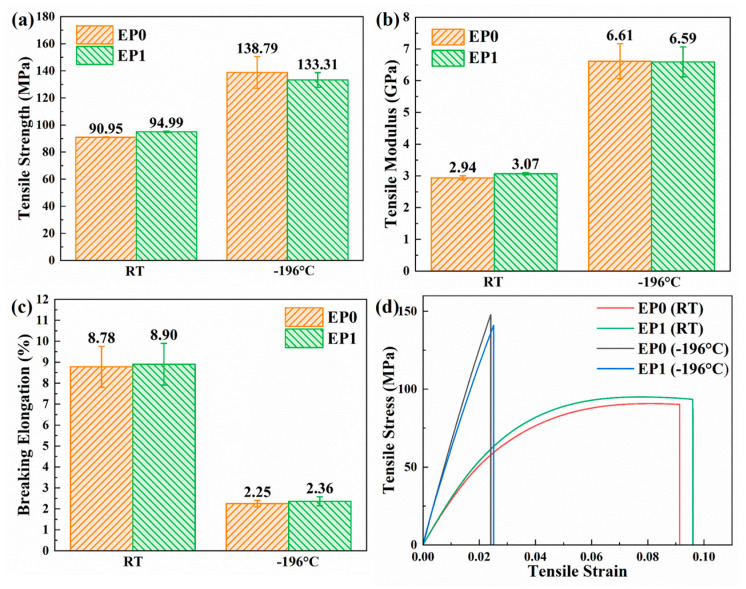
The tensile strength (**a**), tensile modulus (**b**), breaking elongation (**c**), and tensile stress vs. tensile strain curves (**d**) of EP0 and EP1 at RT and −196 °C.

Considering the environment in which the composite liquid oxygen tanks were used, the mechanical properties of EP0 and EP1 at RT and −196 °C were investigated in this paper. The results of the tensile experiment are shown in Figure 9.

**Figure 10:**
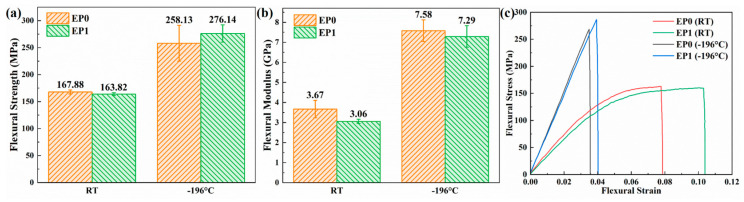
The flexural strength (**a**), flexural modulus (**b**), and flexural stress vs. flexural strain curves (**c**) of EP0 and EP1 at RT and −196 °C.

The tensile strength, tensile modulus, and the breaking elongation of EP1 at RT were 94.99 MPa, 3.07 GPa, and 8.90%, respectively, which were all slightly higher than those of EP0. At −196 °C, the tensile strength of EP1 was 133.31 MPa, which was 3.95% lower than that of EP0; the tensile modulus of EP1 was 6.59 GPa, which is similar to that of EP0; and the breaking elongation of EP1 was 2.36%, which was 4.89% higher than that of EP0, respectively. The flexural properties of EP0 and EP1 were evaluated by three-point loading configuration at RT and at −196 °C. The results of the flexural properties are shown in Figure 10. The flexural strength and flexural modulus of EP1 at RT were 163.82 MPa and 3.06 GPa, which were 2.42% and 16.62% lower than those of EP0, respectively. And at −196 °C, the flexural strength and flexural modulus of EP1 were 276.14 MPa and 7.29 GPa, which, respectively, were 6.98% higher than that of EP0 and 3.83% lower than that of EP0.

Compared with EP0, the enhancement or reduction of most mechanical properties of EP1 was within 5%, except for the significant reduction of flexural modulus at RT and the enhancement of flexural strength at −196°C. Therefore, the addition of TFEPA had small effects on the mechanical properties of the epoxy matrix in general. In addition, the tensile strength, tensile modulus, flexural strength, and flexural modulus of the both epoxy matrixes at −196 °C were significantly higher than those at RT. And the breaking elongation at −196 °C was significantly lower than at RT. This can be explained by the free volume theory. According to the free volume theory, the molecular chains will shrink and freeze with the decrease in temperature, which leads to the strengthening of hydrogen bonding and van der Waals force, ultimately resulting in a significant increase in strength and modulus and a decrease in breaking elongation [52]. In general, the area under the stress vs. strain curve represents the strain energy at breaking, which reflects the toughness of the material [17,53]. As shown in Figure 9d and Figure 10c and Table 6, the strain energy at tensile breaking of EP1 at RT is 7.151 MJ/m^3^, the strain energy at flexural breaking is 12.011 MJ/m^3^, the strain energy at tensile breaking of EP1 at −196 °C is 1.877 MJ/m^3^, and the strain energy at flexural breaking is 5.926 MJ/m^3^. The strain energies at breaking of EP1 at RT and −196 °C were increased by 1.08–38.12% compared to EP0, indicating that the toughness of EP1 was better than EP0. Additionally, the strain energy at breaking of the both resin matrixes at −196 °C was lower than that at RT, indicating that the low temperature toughness of the resin matrixes were lower than the RT toughness. Compared with strength and modulus, the mechanical property requirements of epoxy-based materials for composite fuel tanks are more concerned with toughness, especially low-temperature toughness. EP1 with similar strength and better toughness was more suitable than EP0 as a matrix material for composite liquid oxygen tanks.

### 3.7. Flexural Properties of the CF/EP Composites

**Figure 11:**
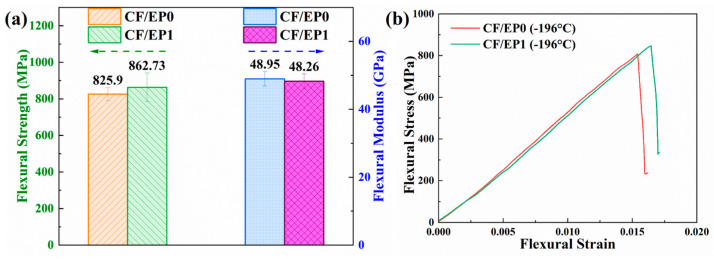
The flexural strength and modulus (**a**) and flexural stress vs. flexural strain curves (**b**) of CF/EP0 and CF/EP1 at −196 °C.

The flexural properties of the CF/EP composites were tested, and the results are shown in Figure 11. The flexural strength of CF/EP1 at −196 °C was 862.73 MPa, which is 4.46% higher than that of CF/EP0. The flexural modulus of CF/EP1 at −196 °C was 48.26 GPa, which is 1.41% lower than that of CF/EP0. It can be seen that the addition of TFEPA slightly enhanced the strength and decreased the modulus of the composite. Similarly, it can be concluded from Figure 11b that the toughness of CF/EP1 at −196 °C was 6.88 MJ/m^3^, which is 10.79% higher than the 6.21 MJ/m^3^ toughness of CF/EP0. This is mainly due to the fact that the EP1 resin exhibited a better toughness at −196 °C than EP0, which improved the interlaminar strength of the composites [54]. In brief, CF/EP1 was more suitable for the manufacture of composite liquid oxygen tanks than CF/EP0.

## 4. Conclusions

In this study, a novel epoxy resin of fluorinated glycidyl amine, TFEPA monomer, was synthesized successfully. The TFEPA monomer was a flowable liquid with a viscosity of about 260 mPa·s from RT to 150 °C. In addition, four resin systems, EP0, EP1, EP2, and EP3, were designed and tested for the viscosity requirements of the filament winding process. The processing properties of EP1, EP2, and EP3 (all with the addition of TFEPA) were significantly better than those of EP0. The viscosity of EP1, EP2, and EP3 remained around 300 mPa·s over a long temperature range. The viscosity of EP0 at 45 °C was 4925 mPa·s, which is not suitable for the filament winding process. With the addition of TFEPA, the viscosity of the resin system was reduced to 558–749 mPa·s and met the requirements of this process. The processing temperature window and processing time for the filament winding process were increased by 73–85% and 186–231%, respectively. Moreover, there was a small difference in processing performance between EP1, EP2, and EP3 with different contents of TFEPA. The curing kinetics of FEP, EP0, and EP1 were analyzed to determine their curing process with DDM, which provided some references for the processing method of the resins. After being cured with DDM, the crosslinked FEP, EP0, and EP1 passed the ASTM G86-17 test, indicating that they were compatible with liquid oxygen. It was proved that addition of TFEPA had no significant effect on the compatibility of liquid oxygen or the thermal properties of the resin system. And the tensile strength, tensile modulus, and breaking elongation of EP1 at −196 °C were 133.31 MPa, 6.59 GPa, and 2.36%, respectively, which were not significantly different from EP0. The flexural strength and modulus were 276.14 MPa and 7.29 GPa at −196 °C, respectively, which were 6.98% higher and 3.83% lower than those of EP0. The strain energy at flexural breaking of EP1 at −196 °C was 5.926 MJ/m^3^, which increased by 21.73% compared to EP0, indicating that the toughness was improved with the addition of TFEPA to the resin system. Finally, composites were prepared by using EP0 and EP1 for the matrix, and the flexural properties of the composites were analyzed. The flexural strength and toughness of composite CF/EP1 at −196 °C were 862.73 MPa and 6.88 MJ/m^3^, which were 4.46% and 10.79% higher than those of CF/EP0. The results showed that the addition of TFEPA improved the flexural properties of the composites, which is beneficial for the manufacture of composite liquid oxygen cryogenic tanks. These results indicate that the fluorinated epoxy resin has easy processing properties and is compatible with liquid oxygen, which can be used as a reactive diluent of the epoxy resin system to improve its processability. More importantly, it can preserve the liquid oxygen compatibility and cryogenic mechanical properties of the original phosphorus-containing epoxy resin systems simultaneously. It is expected that this fluorinated epoxy resin has the potential to be applied in the manufacture of composite liquid oxygen cryogenic tanks.

## Figures and Tables

**Table 1:** Rheological properties of different epoxy matrixes for filament winding process.

Sample	Processing Onset Temperature (°C)	Processing Termination Temperature (°C)	Processing Temperature Window (°C)	Processing Time at 50 °C (min)
EP0	62	103	41	29
EP1	44	115	71	83
EP2	42	116	74	91
EP3	41	117	76	96

**Table 2:** DSC characteristic temperatures of samples at different heating rates.

Sample	Heating Rate (°C/min)	*T_o_* (°C)	*T_p_* (°C)	*T_e_* (°C)
FEP	5	125	161.93	191.3
	10	138.32	176.12	204.15
	15	151.97	187.85	217.51
	20	164.96	199.76	228.17
EP0	5	92.6	135.23	175.83
	10	101.04	150.67	195.58
	15	107.56	158.13	206.31
	20	113.07	165.44	212.48
EP1	5	91.56	142.4	186.62
	10	97.2	153.56	200.3
	15	103.26	164.51	213.23
	20	109.32	174.64	224.97

**Table 3:** The compatibility results of FEP, EP0, and EP1 samples with liquid oxygen.

Sample	Total Number of Test Samples	Burning	Explosion	Flash	Charring	*IRS* (%)
FEP	60	0	0	1	0	1
EP0	20	0	0	0	0	0
EP1	20	0	0	0	0	0
E59 ^a^	20	2	0	1	2	17

^a^ The unmodified DGEBF resin, as control.

**Table 4:** TGA and DTG properties of FEP, EP0, and EP1 samples.

Sample	Heating Rate (°C/min)	T_d5%_ (°C)	T_d10%_ (°C)	T_dmax_ (°C)	Maximum Degradation Rate (%/°C)	Carbon Residue Rate at 800 °C (%)
FEP	5	243	268	385	−1.16	13.04
	10	253	283	401	−1.21	10.72
	15	275	301	414	−1.16	10.68
	20	281	307	421	−1.11	10.97
EP0	5	338	348	361	−1.56	26.46
	10	347	361	377	−1.54	23.00
	15	361	373	389	−1.50	22.43
	20	371	382	398	−1.44	22.21
EP1	5	335	349	366	−1.70	25.03
	10	348	364	380	−1.62	22.55
	15	360	374	390	−1.58	21.71
	20	373	385	401	−1.52	21.39

**Table 5:** DSC and DMA results of FEP, EP0, and EP1 samples.

Sample	*T_g_*_(DSC)_ ^a^ (°C)	*T_g_*_(DMA)_ ^b^ (°C)	*E*′ (MPa)	$E_{r}^{\prime }$ (MPa)	ρ (mol/m^3^)
FEP	108	128	2680.86	7.21	671
EP0	148	159	2247.01	23.21	2014
EP1	144	154	2186.11	22.86	2006

^a^ The *T_g_* (DSC) was obtained from DSC curves. ^b^ The *T_g_* (DMA) was obtained from the peak of the tan δ vs. temperature curves.

**Table 6:** Tensile and flexural strain energy at breaking of EP0 and EP1 at RT and −196 °C.

Temperature	Sample	Tensile Strain Energy (MJ/m^3^)	Relative Percentage ^a^ (%)	Flexural Strain Energy (MJ/m^3^)	Relative Percentage (%)
RT	EP0	6.343	-	8.696	-
	EP1	7.151	+12.74	12.011	+38.12
−196 °C	EP0	1.857	-	4.868	-
	EP1	1.877	+1.08	5.926	+21.73

^a^ Relative percentage is the ratio of EP1 to EP0 at the same temperature.

## Data Availability

The original contributions presented in the study are included in the article/Supplementary Materials; further inquiries can be directed to the corresponding authors.

## Supplementary Materials

The following supporting information can be downloaded at: https://www.mdpi.com/article/10.3390/polym16192759/s1, Figure S1: Viscosity of TFEPA monomer; Figure S2: Characteristic temperature fitting curves of FEP at different heating rates; Figure S3: Characteristic temperature fitting curves of EP0 at different heating rates; Figure S4: Characteristic temperature fitting curves of EP1 at different heating rates; Figure S5: Linear fit of $\ln\left(\beta /T_{p}^{2}\right)$ vs. 1/$T_{p}$ of FEP; Figure S6: Linear fit of ln⁡(β) vs. 1/$T_{p}$ of FEP; Figure S7: Linear fit of $\ln\left(\beta /T_{p}^{2}\right)$ vs. 1/$T_{p}$ of EP0; Figure S8: Linear fit of ln⁡(β) vs. 1/$T_{p}$ of EP0; Figure S9: Linear fit of $\ln\left(\beta /T_{p}^{2}\right)$ vs. 1/$T_{p}$ of EP1; Figure S10: Linear fit of ln⁡(β) vs. 1/$T_{p}$ of EP1.
