# Supplementary material for: Preparation and Application of a Novel Liquid Oxygen-Compatible Epoxy Resin of Fluorinated Glycidyl Amine with Low Viscosity
Source: Polymers (Basel). 2024 Sep 29;16(19):2759. doi: 10.3390/polym16192759 (PMC11478847; doi:10.3390/polym16192759)
Supplement: Supplementary file 1 [file polymers-16-02759-s001.zip › polymers-3202870-supplementary.pdf]

Supplementary Materials

Preparation and Application of a Novel Liquid Oxygen-Compatible Epoxy Resin of Fluorinated Glycidyl Amine with Low Viscosity

Jianing Wei [1], Jia Yan [2,*], Shichao Li [3], Juanzi Li [2] and Zhanjun Wu [2,*]

[1] School of Mechanics and Aerospace Engineering, Dalian University of Technology, Dalian 116024, China; conscript@mail.dlut.edu.cn
[2] School of Materials Science and Engineering, Dalian University of Technology, Dalian 116024, China; jzl1101@mail.dlut.edu.cn
[3] School of Fiber Engineering and Equipment Technology, Jiangnan University, Wuxi 214122, China; lsc1212@jiangnan.edu.cn
* Correspondence: jyan@dlut.edu.cn (J.Y.); wuzhj@dlut.edu.cn (Z.W.)

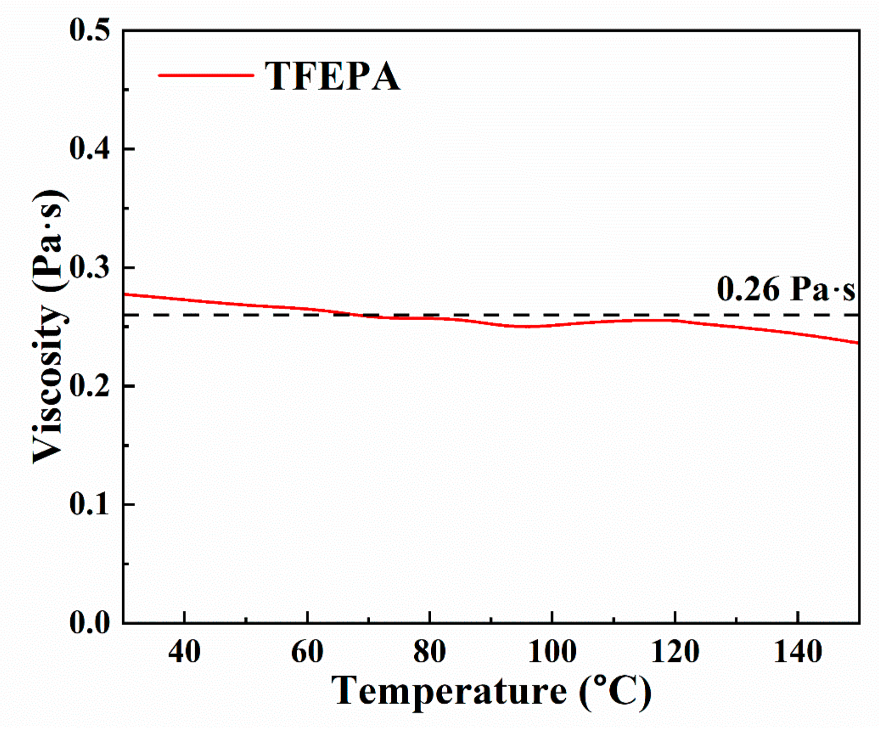

Figure S1. Viscosity of TFEPA monomer.

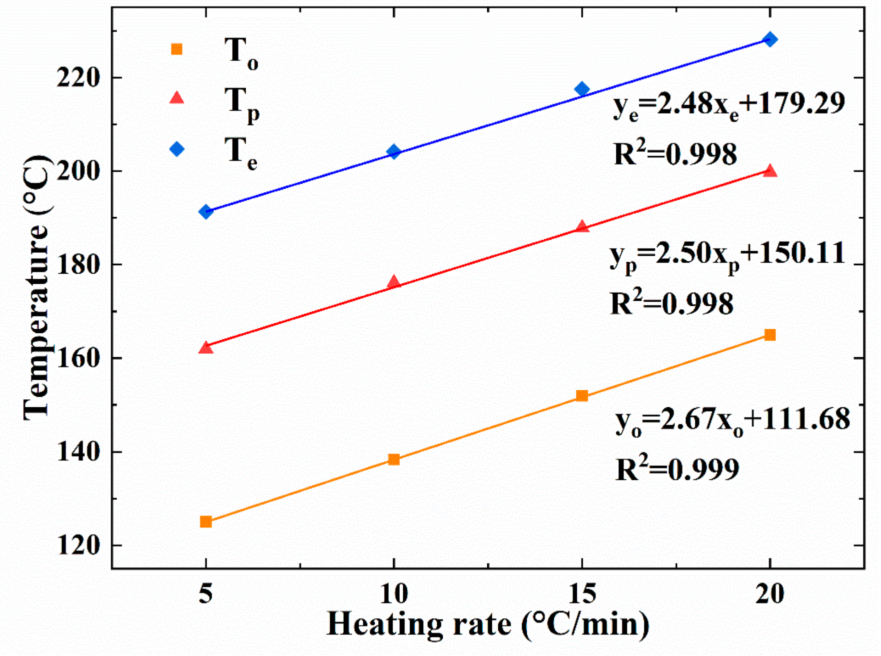

Figure S2. Characteristic temperature fitting curves of FEP at different heating rates.

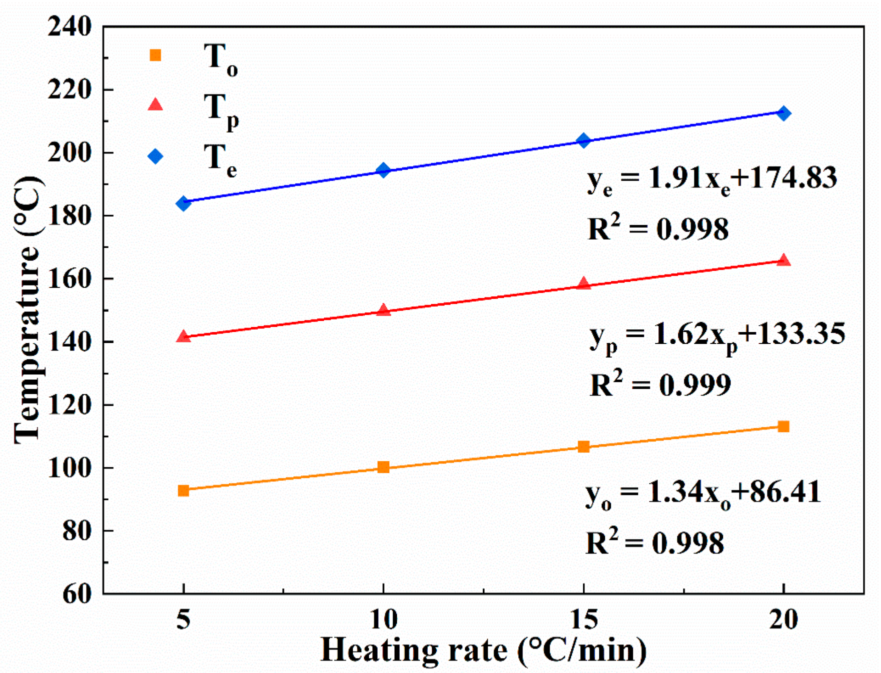

Figure S3. Characteristic temperature fitting curves of EP0 at different heating rates.

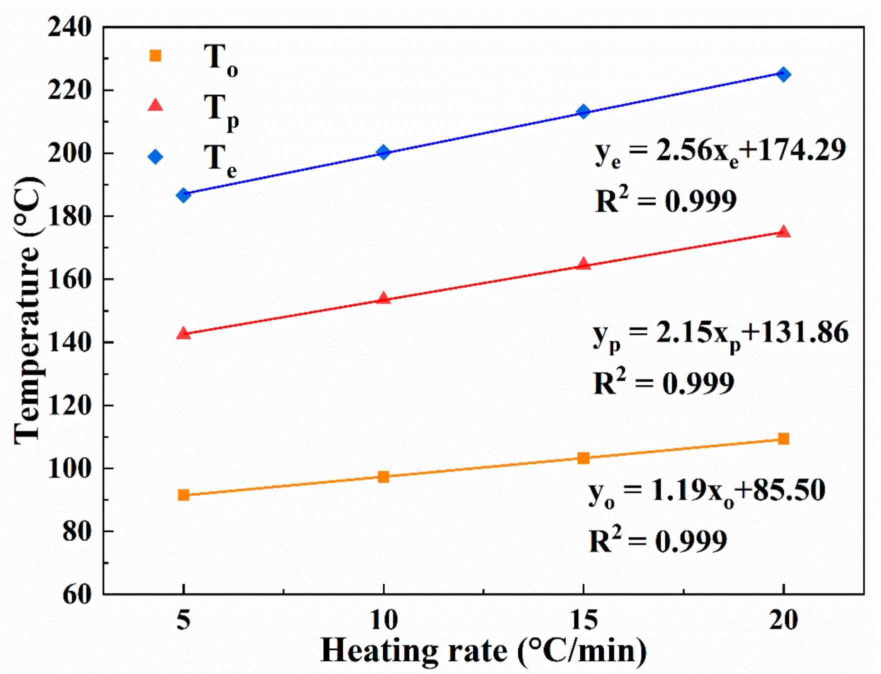

Figure S4. Characteristic temperature fitting curves of EP1 at different heating rates.

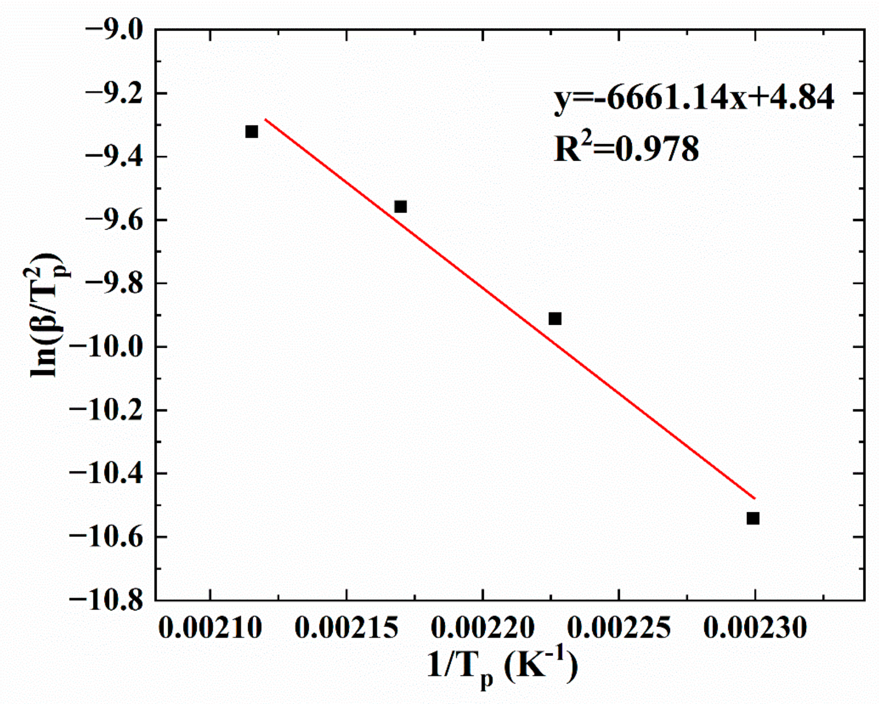

Figure S5. Linear fit of $\ln(\beta/T_p^2)$ vs. $1/T_p$ of FEP.

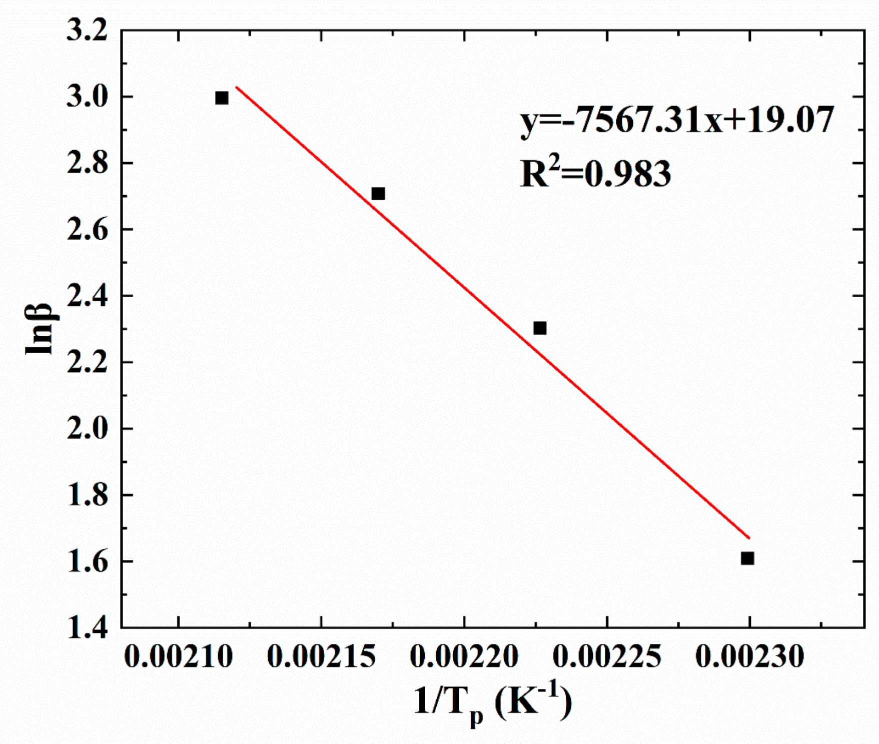

Figure S6. Linear fit of $\ln(\beta)$ vs. $1/T_p$ of FEP.

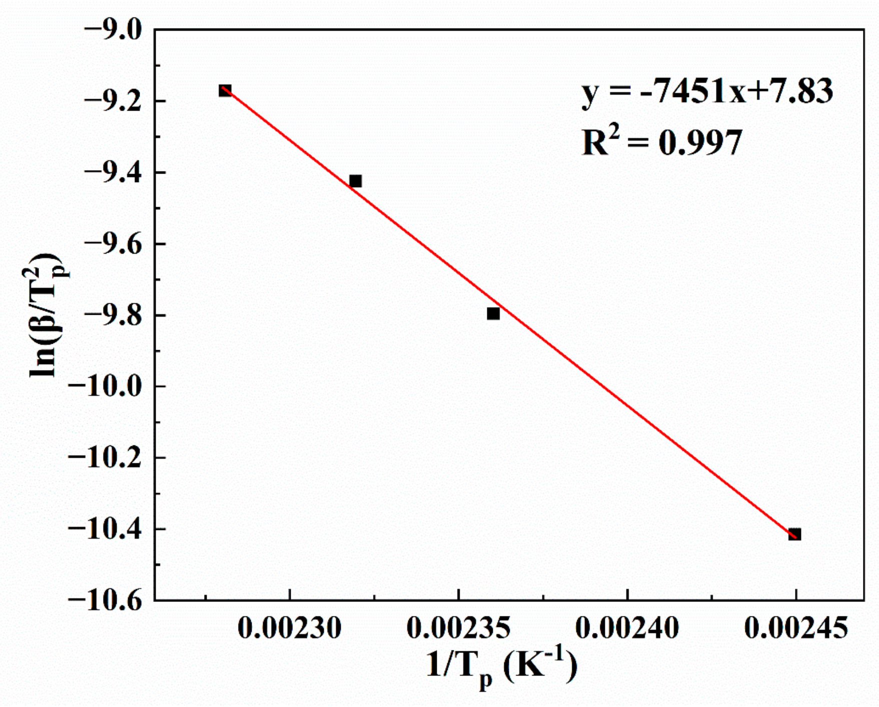

Figure S7. Linear fit of $\ln(\beta/T_p^2)$ vs. $1/T_p$ of EP0.

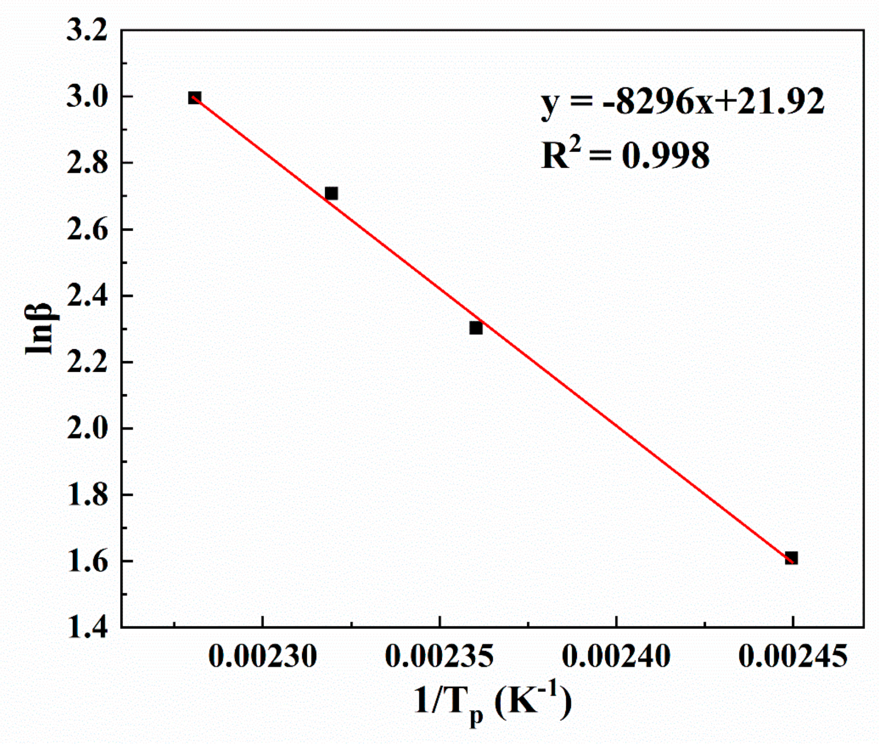

Figure S8. Linear fit of $\ln(\beta)$ vs. $1/T_p$ of EP0.

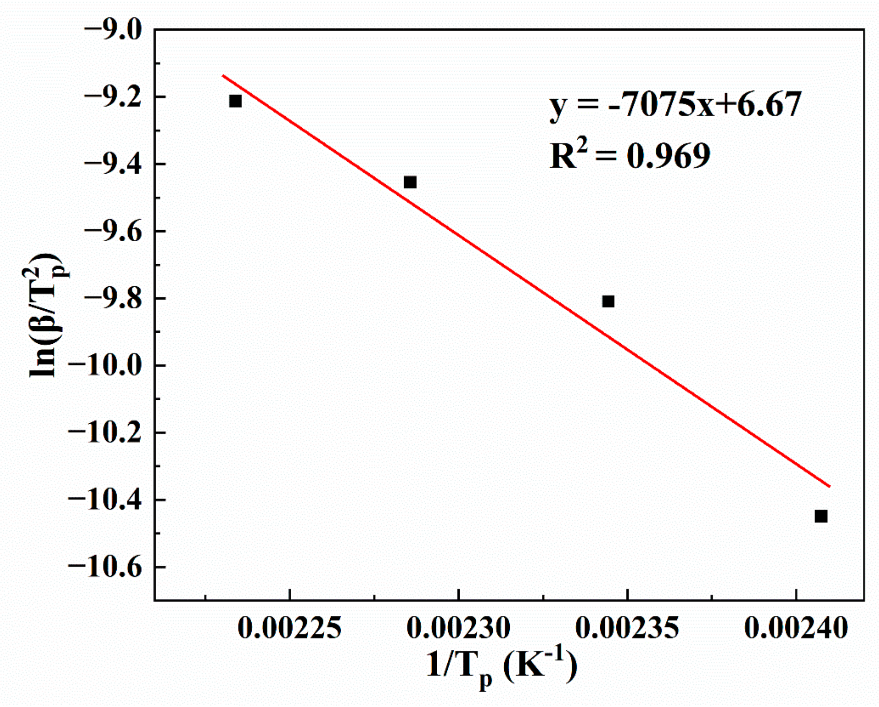

Figure S9. Linear fit of $\ln(\beta/T_p^2)$ vs. $1/T_p$ of EP1.

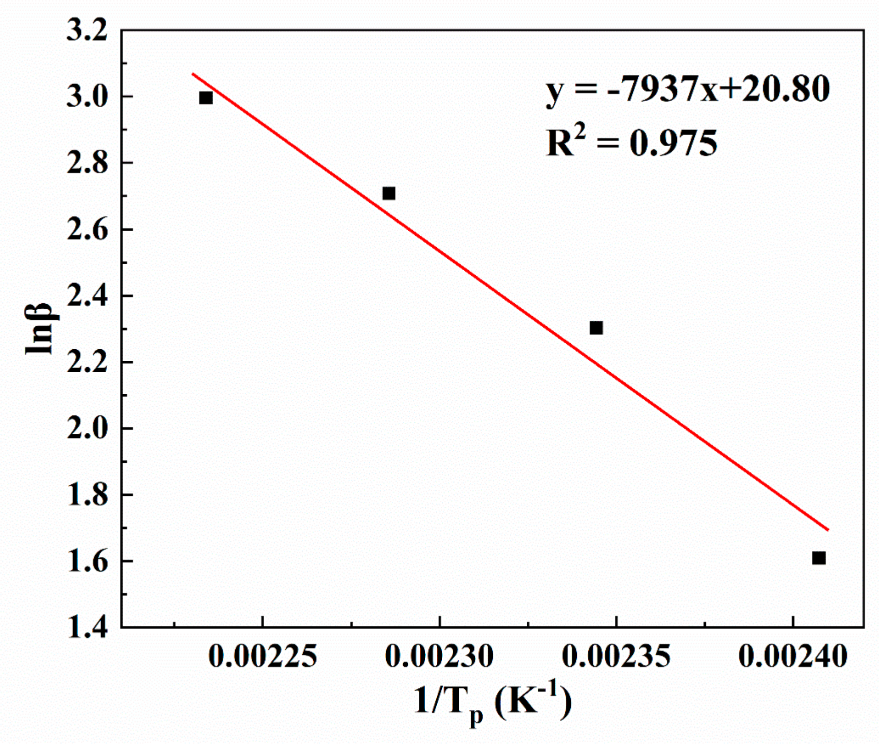

Figure S10. Linear fit of $\ln(\beta)$ vs. $1/T_p$ of EP1.
